# Dose-Dependent Intestinal Transcriptomic and Metabolomic Responses to Acute Waterborne Glyphosate Exposure in the Sea Cucumber (*Apostichopus japonicus*)

**DOI:** 10.3390/biology15090694

**Published:** 2026-04-28

**Authors:** Jingchun Sun, Libing Zhang, Christopher D. Hepburn, Shaoping Kuang, Hongsheng Yang

**Affiliations:** 1 College of Environment and Safety Engineering, Qingdao University of Science and Technology, Qingdao 266042, China; sunjingchun@qdio.ac.cn; 2 Laboratory of Marine Ecology and Environmental Sciences, Institute of Oceanology, Chinese Academy of Sciences, Qingdao 266000, China; zhanglibin@qdio.ac.cn; 3 Laboratory for Marine Ecology and Environmental Science, Qingdao Marine Science and Technology Center, Qingdao 266237, China; 4 State Key Laboratory of Breeding Biotechnology and Sustainable Aquaculture, Institute of Oceanology, Chinese Academy of Sciences, Qingdao 266000, China; 5 Department of Marine Science, University of Otago, Dunedin 9054, New Zealand; chris.hepburn@otago.ac.nz

**Keywords:** glyphosate, *Apostichopus japonicus*, acute waterborne exposure, transcriptomics, metabolomics, intestinal response, dose-dependent toxicity, marine invertebrate

## Abstract

Glyphosate is one of the most widely used herbicides worldwide and has been increasingly reported in aquatic environments, including riverine, estuarine, and coastal systems. However, information on its intestinal effects in benthic marine invertebrates remains limited. In this study, we investigated dose-dependent intestinal responses of the sea cucumber *Apostichopus japonicus* following acute waterborne glyphosate exposure using integrated transcriptomic and metabolomic analyses. Sea cucumbers were exposed for 24 h to four nominal glyphosate concentrations: 0, 9.23, 46.15, and 230.77 mg/L. Mortality occurred only in the highest-concentration group, allowing phenotypic stratification of this group into high-dose survivors (HL) and high-dose dead individuals (HD) for downstream multi-omics comparisons. Principal component analysis and orthogonal partial least-squares discriminant analysis indicated clear exposure- and phenotype-associated shifts in intestinal molecular profiles. Differential expression analysis and pathway enrichment showed that low-dose exposure was mainly associated with metabolic and digestion-related adjustments, whereas higher exposure levels were characterized by broader perturbation of immune regulation, stress-response signaling, proteostasis-related processes, and cell fate-associated pathways. Metabolomic profiling further revealed progressive remodeling of lipid, amino acid, energy, redox, and transport-related pathways, with the most extensive alterations observed in HD. Integrated transcriptome–metabolome analysis supported increasingly structured cross-omics covariation with rising exposure severity, highlighting coordinated intestinal system disruption under high-dose glyphosate stress. Overall, these findings demonstrate that acute waterborne glyphosate exposure induces dose-dependent intestinal molecular reprogramming in *A. japonicus*, with marked divergence between surviving and dead individuals at the highest exposure level. This study provides mechanistic evidence for early intestinal responses to glyphosate in a representative marine deposit-feeding invertebrate and offers a basis for future studies linking controlled exposure experiments with environmentally relevant marine risk scenarios.

## Simple Summary

Glyphosate is a widely used herbicide that can enter coastal waters through runoff, but its effects on the gut of benthic marine animals remain poorly understood. Sea cucumbers live on the seafloor and feed on sediment-associated material, making them a useful model for studying intestinal responses to waterborne pollutants. In this study, we exposed sea cucumbers to increasing concentrations of glyphosate in seawater for 24 h and then examined changes in intestinal metabolites and gene expression. Mortality occurred only at the highest exposure level, allowing us to compare surviving and dead individuals within the same treatment. We found clear dose-dependent molecular changes in the intestine, with the strongest disruption in dead individuals and an intermediate pattern in survivors. These changes were associated with altered energy and nutrient metabolism, lipid and amino acid pathways, and stress-related responses. Our results provide new mechanistic insight into how acute glyphosate exposure affects intestinal function in a marine deposit-feeding invertebrate and offer a foundation for future studies under more environmentally relevant conditions.

## 1. Introduction

Sea cucumbers are ecologically important deposit-feeding invertebrates that contribute to sediment bioturbation and organic matter recycling, while also supporting high-value aquaculture in East Asia and other coastal regions [1,2]. Among farmed holothurians, *Apostichopus japonicus* is a representative species in which intestinal physiology and gut-associated microbiota are closely associated with growth performance, health status, and responses to environmental stress [3,4,5,6]. In deposit-feeding species, the intestine functions not only as a digestive organ but also as a key interface where sediment-associated microorganisms and metabolites interact with host physiological processes, including epithelial barrier function, immune regulation, and redox homeostasis [4,6,7,8,9]. Accordingly, gut-focused omics approaches have become increasingly useful for dissecting stress responses in sea cucumbers by linking changes in host transcriptional regulation, metabolic profiles, and, where relevant, intestinal microbiota under environmental challenges.

Herbicide contamination is an emerging concern for coastal and estuarine mariculture because riverine transport and surface runoff can carry polar pesticides into nearshore waters and sediments, thereby creating plausible exposure scenarios for benthic and deposit-feeding species [10,11,12]. Glyphosate (N-(phosphonomethyl)glycine) and its major metabolite, aminomethylphosphonic acid (AMPA), have been widely reported in freshwater and transitional aquatic systems and are increasingly considered relevant contaminants in river-to-coast transport pathways [10,11,13]. Recent river-to-sea and watershed-scale studies have reported measurable glyphosate and AMPA in water and sediments, supporting the plausibility of coastal inputs and sediment-associated exposure routes for marine benthic organisms [11,13]. Importantly, analytical challenges associated with high-salinity matrices have historically limited direct marine measurements, although recently developed seawater- or salinity-adapted methods are improving the detection of glyphosate and AMPA in saline environments [12]. Together, these findings highlight the need to evaluate sublethal, mechanism-centered endpoints in marine invertebrates while recognizing that measured glyphosate data in fully marine settings remain comparatively limited and that controlled exposure studies are therefore still important for defining early-response mechanisms [10,11,14].

A defining mechanistic feature of glyphosate is its inhibition of 5-enolpyruvylshikimate-3-phosphate synthase (EPSPS) in the shikimate pathway, a pathway absent in metazoans but essential in plants, fungi, and many microorganisms [15,16]. This feature provides a biologically plausible route by which glyphosate may indirectly affect animal hosts through microbiome-mediated changes in aromatic amino acid metabolism, microbial community composition, and downstream host–microbe interactions [15,16,17,18]. Indeed, glyphosate or glyphosate-based herbicides have been associated with behavioral and physiological disturbances in fish models, and separate studies have also reported exposure-related alterations in intestinal microbiota composition, suggesting that dysbiosis and metabolic disruption may accompany glyphosate exposure under some conditions [17,19]. Chronic glyphosate-based herbicide exposure has also been shown to perturb gill- and gut-associated microbial communities in rainbow trout, reinforcing that microbiome-related endpoints can be sensitive indicators in aquatic vertebrates [20]. Beyond microbiota structure, aquatic toxicology research increasingly emphasizes pathway-level responses, including oxidative stress, epithelial barrier function, immune regulation, and energy metabolism, because these processes often translate chemical exposure into organism-level effects [10,14,21].

In sea cucumbers, the intestine is a major target tissue for diverse environmental stressors, and recent omics-based studies have begun to reveal recurring patterns of gut injury, metabolic disturbance, and homeostatic disruption. For example, sulfide exposure induces histological damage together with oxidative stress, metabolic disorders, and gut microbiota dysbiosis in juvenile *A. japonicus* [22]. Nanoplastics and other emerging stressors can likewise reshape gut microbial assembly and are associated with altered physiological status in sea cucumbers, underscoring the importance of intestinal homeostasis in stress tolerance [23,24]. Gut microbiota–metabolite coupling has also been implicated in the aestivation physiology of *A. japonicus*, suggesting that intestinal metabolites can provide interpretable functional readouts of host–microbe interactions under thermal challenge [9]. At the host regulatory level, heat stress responses in *A. japonicus* involve epigenetic and transcriptional remodeling, highlighting that environmental stress can propagate from signaling and chromatin regulation to downstream metabolic responses [25]. Despite these advances, glyphosate-specific evidence in holothurians remains scarce, particularly at the level of intestinal transcriptomic–metabolomic integration, and it remains unclear whether acute waterborne glyphosate exposure induces dose-dependent, coordinated changes in transcriptional networks and metabolite profiles that can be integrated into a coherent intestinal response framework. Addressing such questions requires integrative analytical frameworks that move beyond parallel lists of differentially expressed genes (DEGs) and differential metabolites (DAMs) toward analyses that identify shared patterns of variation and biologically interpretable response axes across omics layers [26,27,28]. Network-based and module-based approaches can help summarize transcriptomic variation into coordinated biological patterns associated with exposure gradients or phenotypes [26]. For functional interpretation, Gene Ontology (GO) and KEGG pathway analyses are widely used to summarize transcriptomic and metabolomic changes at the pathway level, and these approaches can be complemented by broader enrichment-based interpretation strategies when subtle but coordinated shifts are of interest [29]. At the multi-omics level, methods such as O2PLS and related latent-variable models can separate shared variation from data-type-specific variation, thereby improving the identification of robust cross-omics associations [27,28]. More broadly, multi-block integration frameworks have been developed to support supervised integration and feature selection across multiple omics layers when discrimination among experimental groups is of interest [30,31]. Importantly, integrative analysis should not stop at statistical association alone but should instead link co-varying genes and metabolites to plausible physiological processes such as redox regulation, epithelial function, immune response, and energy metabolism in order to generate mechanistically meaningful hypotheses [21,32].

In this study, we investigated dose-dependent intestinal transcriptomic and metabolomic responses of A. japonicus under acute waterborne glyphosate exposure. We combined RNA-seq-based transcriptomics with LC–MS-based untargeted metabolomics to characterize exposure-associated changes in (i) transcriptional responses and pathway enrichment, (ii) metabolic reprogramming and pathway-level metabolite shifts, and (iii) cross-omics coordination revealed by integrative and correlation-based analyses. By integrating DEG and differential metabolite evidence with pathway-level interpretation and cross-omics coupling patterns, we aimed to identify interpretable response features associated with glyphosate-triggered intestinal disturbance in a representative marine deposit feeder. This study provides mechanistic insight into early intestinal responses to glyphosate in a mariculture-relevant marine invertebrate and offers a basis for future studies linking controlled exposure experiments with environmentally relevant coastal scenarios [10,11,12,33,34,35].

## 2. Materials and Methods

### 2.1. Chemicals

Glyphosate (N-(phosphonomethyl)glycine; CAS No. 1071-83-6; catalog no. N817057-100 g; Macklin Biochemical Co., Ltd., Shanghai, China; purity 95%) was used in the acute waterborne exposure experiment. Glyphosate was added directly as a powder to the exposure seawater according to the experimental design rather than being prepared as a liquid stock solution.

### 2.2. Animals and Acclimation

Sea cucumbers (*Apostichopus japonicus*; body weight 50–100 g) were randomly selected for the experiment. Before exposure, animals were acclimated under laboratory conditions in filtered seawater. The seawater used for acclimation and exposure was from the same monitored water source and had been confirmed to be free of glyphosate. Water quality parameters, including salinity, pH, and dissolved oxygen, were routinely monitored and maintained within ranges suitable for sea cucumber culture. During acclimation, sea cucumbers were maintained under continuous aeration with a 12 h light:12 h dark photoperiod, and the water was renewed once every 24 h. Sea cucumbers were not fed during the acclimation period.

### 2.3. Acute Waterborne Glyphosate Exposure Design and Phenotypic Stratification at the Endpoint

Sea cucumbers were randomly assigned to four treatment groups: a control group (C), a low-concentration glyphosate treatment group (L), a medium-concentration glyphosate treatment group (M), and a high-concentration glyphosate treatment group (H). Each treatment consisted of three independent culture buckets, with 10 sea cucumbers in each bucket, giving a total of 30 individuals per treatment group.

Exposure was conducted in the same monitored filtered seawater used during acclimation, under continuous aeration and a 12 h light:12 h dark photoperiod. The measured glyphosate concentrations in the exposure water were 0 mg/L in C, 9.23 mg/L in L, 46.15 mg/L in M, and 230.77 mg/L in H, and group assignment was based on these measured concentrations. The highest exposure concentration was selected with reference to previous studies indicating that this level approaches an acute median lethal range, whereas the 24 h exposure duration was chosen to characterize early molecular responses under acute glyphosate stress. This design also allowed clear phenotypic differentiation at the highest concentration, where surviving and dead individuals exhibited distinct external features. In the high-concentration group, dead individuals showed obvious symptoms such as body wall softening/sloughing, tentacle contraction, and body wall rigidity, consistent with the acute toxic phenotype described in our previous study.

Mortality occurred only in the high-concentration treatment. Therefore, for downstream omics analyses, individuals in the high-concentration group were further stratified into a high-dose survivor group (HL) and a high-dose dead group (HD) according to their endpoint phenotype. This phenotype-based stratification was applied only to endpoint comparative analyses and did not alter the original experimental design of four treatment groups (C, L, M, and H).

### 2.4. Sampling Strategy and Sample Sizes for Multi-Omics

At the 24 h endpoint, 10 sea cucumbers were randomly sampled from each treatment group and labeled individually as No. 1–10. Samples were not pooled, and each sea cucumber was treated as an independent biological individual. Sampling in each treatment group covered all parallel culture buckets to minimize bucket-specific bias.

For transcriptomics (RNA-seq), three biological replicates per analysis group were used (*n* = 3). Whenever possible, RNA-seq replicates were selected such that each replicate originated from a different culture bucket to preserve biological independence. For metabolomics, six biological replicates per analysis group were used (*n* = 6). Consequently, the omics analyses comprised five analysis groups: C, L, M, HL, and HD. For glyphosate residue determination, the sample size was *n* = 3. To minimize post-mortem artifacts in the HD subgroup, intestinal tissues were dissected immediately at the 24 h endpoint and snap-frozen in liquid nitrogen within 1 min after endpoint assessment. Only samples meeting RNA quality requirements were used for RNA-seq analysis.

### 2.5. RNA Extraction, mRNA Library Construction, and Sequencing

Total RNA was extracted from intestinal tissue using a TRIzol-based protocol. RNA quality was assessed by agarose gel electrophoresis, NanoDrop spectrophotometry (Thermo Fisher Scientific, Waltham, MA, USA), Qubit 4.0 fluorometric quantification (Thermo Fisher Scientific), and Agilent 2100 Bioanalyzer analysis (Agilent Technologies, Santa Clara, CA, USA). For eukaryotic transcriptome library construction, poly(A)-containing mRNA was enriched using oligo(dT)-coupled magnetic beads. The enriched mRNA was then fragmented in buffer and used as the template for cDNA synthesis. According to the library construction workflow, mRNA fragmentation was performed in Frag/Prime Buffer at 94 °C for 5 min prior to first-strand cDNA synthesis. First-strand cDNA was synthesized using random oligonucleotide primers, followed by second-strand cDNA synthesis. The purified double-stranded cDNA then underwent end repair, A-tailing, adapter ligation, PCR amplification, and bead-based purification to generate the final sequencing library.

cDNA libraries were prepared using the Hieff NGS^®^ Ultima Dual-mode mRNA Library Prep Kit (12309ES, Yeasen, Shanghai, China). After adapter ligation, libraries were PCR-amplified under the following conditions: 98 °C for 1 min; 12 cycles of 98 °C for 10 s and 60 °C for 75 s; 72 °C for 30 s; and a final extension at 72 °C for 5 min. Amplified libraries were purified using Hieff NGS^®^ DNA Selection Beads (Yeasen Biotechnology Co., Ltd., Shanghai, China), and library quality was evaluated using the Agilent DNA 1000 assay kit or High Sensitivity DNA assay kit, as appropriate. Library construction and sequencing were carried out by Gene Denovo (Guangzhou, China) on an Illumina sequencing platform (Illumina, San Diego, CA, USA).

### 2.6. Transcriptome Read Processing, Mapping, Quantification, Differential Expression, and PCA

Raw sequencing reads were quality-filtered using fastp v0.23.2 to remove reads containing adapter contamination, reads with >10% ambiguous bases (N), reads composed entirely of adenine bases, and low-quality reads in which bases with Q ≤ 20 accounted for more than 50% of the read length. The resulting clean reads were first aligned to the ribosomal RNA database using Bowtie2 v2.4.5 under a no-mismatch setting to remove residual rRNA-derived reads. The remaining unmapped reads were then aligned to the *Apostichopus japonicus* reference genome (NCBI accession ASM275485v1) using HISAT2 v2.2.1, a splice-aware aligner suitable for RNA-seq data. Based on the HISAT2 alignment results, transcripts were reconstructed using StringTie v2.2.1, and gene expression levels were quantified using RSEM v1.3.3. Expression abundance was presented as both raw read counts and fragments per kilobase of transcript per million mapped reads (FPKM).

Differential expression analysis was performed on raw count matrices using DESeq2 v1.36.0 in R. Genes with |log2FC| > 1 and a Benjamini–Hochberg false discovery rate (FDR)-adjusted *p*-value < 0.05 were considered differentially expressed. For visualization purposes, normalized expression values and FPKM values were used where appropriate, whereas all formal statistical inference was based on the DESeq2 count-based framework.

Principal component analysis (PCA) was performed using the gmodels v2.18.1 package in R as an unsupervised multivariate analysis. PCA compresses the original high-dimensional dataset into a small number of principal components, among which PC1 explains the most prominent variation in the data matrix and PC2 explains the most significant variation not captured by PC1. This analysis was used to visualize the overall transcriptomic differences among groups and the degree of variation among samples within each group.

### 2.7. Metabolite Extraction for Untargeted LC–MS/MS

Untargeted metabolomics was performed by Gene Denovo (Guangzhou, China). For tissue samples, approximately 100 mg of intestinal tissue was individually ground in liquid nitrogen and extracted with 500 μL of prechilled 80% methanol. After vortexing, samples were incubated on ice for 5 min and centrifuged at 15,000× *g* at 4 °C for 20 min. A portion of the supernatant was diluted with LC–MS-grade water to a final methanol content of 53%, transferred to a fresh tube, and centrifuged again at 15,000× *g* at 4 °C for 20 min. The resulting supernatant was injected into the LC–MS/MS system for analysis. Pooled QC samples were prepared as equal-volume mixtures of all experimental samples for system conditioning and stability monitoring. Blank samples consisting of 53% methanol–water were processed in parallel to remove background ions.

### 2.8. UHPLC–MS/MS Conditions and Metabolomics Data Processing

UHPLC–MS/MS analyses were performed on a Vanquish UHPLC system coupled to an Orbitrap Q Exactive™ HF-X mass spectrometer (Thermo Fisher, Waltham, MA, USA) at Gene Denovo (Guangzhou, China). Samples were injected onto a Hypesil Gold C18 column (100 × 2.1 mm, 1.9 μm) maintained at 40 °C using a 17 min linear gradient at a flow rate of 0.2 mL/min. In positive ion mode, the mobile phases were 0.1% formic acid in water (A) and methanol (B); in negative ion mode, the mobile phases were 5 mM ammonium acetate (pH 9.0) (A) and methanol (B). The mass spectrometer was operated in positive/negative polarity mode over an m/z range of 100–1500 with a spray voltage of 3.5 kV, sheath gas at 35 psi, auxiliary gas at 10 L/min, capillary temperature at 320 °C, S-lens RF level of 60, and auxiliary gas heater temperature at 350 °C. MS/MS acquisition was performed in a data-dependent manner.

Raw LC–MS files (.raw) were converted to mzXML format using ProteoWizard v3.0.20325 and processed using XCMS v3.16.0 for peak extraction, retention-time alignment, and quantification. Metabolite annotation was performed by matching accurate mass information with a 10 ppm mass tolerance together with adduct patterns against a high-quality MS/MS spectral database. Background ions were removed using blank samples, and peak intensity data were normalized according to the service-provider workflow before downstream statistical analysis.

PCA was used as an unsupervised multivariate method to summarize the major trends of metabolomic variation among groups and the degree of within-group dispersion. OPLS-DA was used as a supervised multivariate method to further assess group discrimination. In the permutation validation plots, R2 represents the goodness of fit of the model, and Q2 represents its predictive ability. The T score in the OPLS-DA score plots represents the sample score on the predictive latent component, reflecting the projection of each sample in the discriminant space.

### 2.9. Functional Enrichment and Transcriptome–Metabolome Integration

For transcriptomics, differentially expressed genes (DEGs) were annotated to Gene Ontology (GO) terms and KEGG pathways, and enrichment analyses were performed using standard over-representation methods with multiple-testing correction. For metabolomics, KEGG pathway enrichment was performed based on the annotated differential metabolites derived from the untargeted LC–MS/MS workflow. In this study, q-values refer to false discovery rate (FDR)-adjusted *p*-values.

To summarize joint biological signals across omics layers, all DEGs and differential metabolites were mapped to the KEGG pathway database. KEGG pathway maps provide a framework linking gene functions with endogenous metabolite structures and metabolic pathways, thereby allowing integrated interpretation of transcriptomic and metabolomic changes. Shared KEGG pathways supported by both DEGs and differential metabolites were identified by pathway co-annotation and summarized as joint evidence.

To further integrate transcriptomic and metabolomic datasets, two-way orthogonal partial least-squares (O2PLS) analysis was performed using the OmicsPLS v1.2.0 package in R. This method decomposes the variation in the two data matrices into joint variation shared between datasets, orthogonal variation specific to each dataset, and residual noise. The optimal numbers of joint and orthogonal components were determined using the cross-validation procedure implemented in the package, and the best-fitting model was used for downstream integration analysis.

Pearson correlation analysis was also performed to evaluate transcript–metabolite associations. Gene–metabolite pairs were ranked in descending order according to the absolute Pearson correlation coefficient. The top 50 genes and metabolites were selected for visualization in the correlation heatmap using the pheatmap v1.0.12 package in R. In addition, the top 250 gene–metabolite pairs with an absolute Pearson correlation coefficient >0.5 were used to construct the metabolite–transcript association network using the igraph v1.4.1 package in R.

## 3. Results

### 3.1. Transcriptomic Responses of Apostichopus japonicus to Acute Glyphosate Exposure

#### 3.1.1. Principal Component Analysis (PCA) of Transcriptomic Profiles

**Figure 1:**
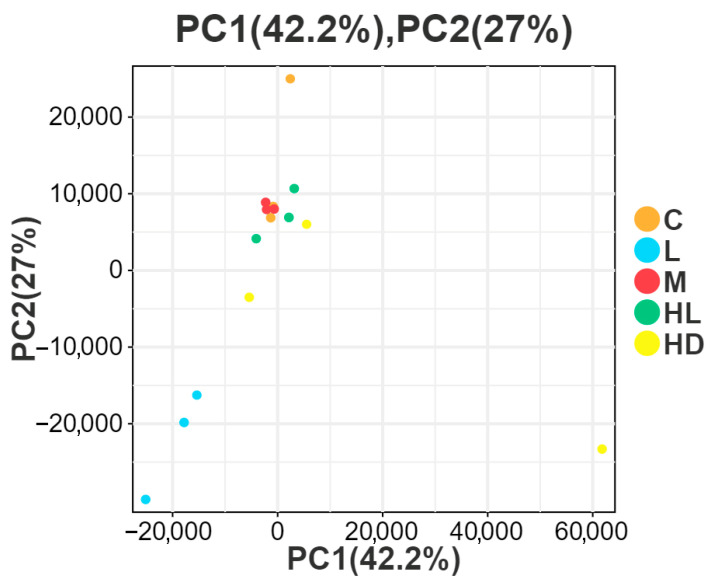
PCA of intestinal transcriptomic profiles across glyphosate exposure groups. Principal component analysis (PCA) was performed using the normalized transcriptomic expression matrix. PC1 and PC2 explained 42.2% and 27.0% of the total variance, respectively. C, control; L, low concentration; M, medium concentration; HL, high-dose survivors; HD, high-dose dead. The PCA plot was used to visualize overall transcriptomic differences among groups and within-group dispersion.

To assess the overall transcriptomic variation associated with acute glyphosate exposure, principal component analysis (PCA) was performed using the normalized expression matrix. PC1 and PC2 explained 42.2% and 27.0% of the total variance, respectively, accounting for 69.2% of the cumulative variation (Figure 1). The PCA score plot showed that the low-concentration group (L) was clearly separated from the control group (C), indicating a distinct transcriptional response under low-dose exposure. By contrast, the medium-concentration group (M) and the high-dose survivor group (HL) showed partial overlap with the control group, suggesting comparatively weaker or less coordinated global transcriptomic shifts. The high-dose dead group (HD) exhibited the greatest within-group dispersion, with several samples deviating markedly from the main cluster. This pattern indicates pronounced heterogeneity in the lethal phenotype and was therefore taken into account in downstream pathway-level interpretation rather than being overinterpreted at the level of global clustering alone.

Therefore, Figure 1 should be interpreted as an overview of global transcriptomic structure rather than as evidence of complete group-wise separation, and the biological relevance of the HD distribution was considered together with downstream functional enrichment and cross-omics integration results.

#### 3.1.2. Differential Gene Expression Analysis

**Figure 2:**
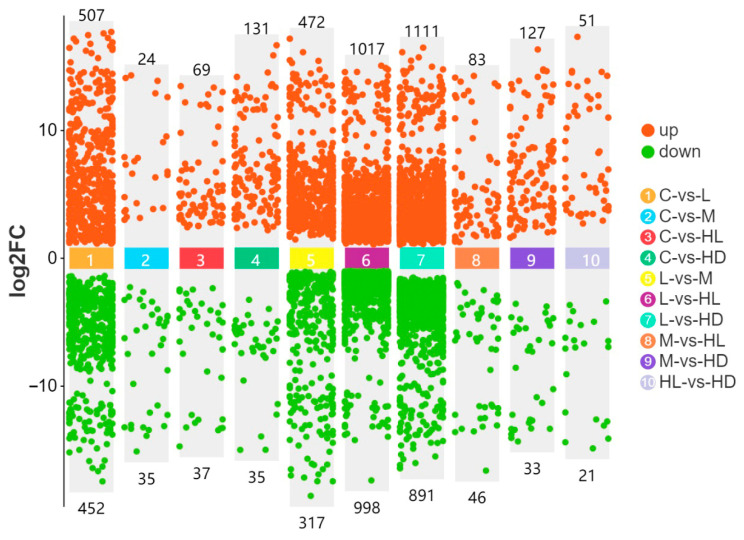
Numbers of differentially expressed genes (DEGs) in control-based comparisons. The numbers of upregulated and downregulated genes are shown for the four main comparisons: C vs. L, C vs. M, C vs. HL, and C vs. HD. Differentially expressed genes were defined as genes with |log2FC| > 1 and an FDR-adjusted *p*-value < 0.05.

Differential expression analysis revealed transcriptional responses to glyphosate exposure in all control-based comparisons (Figure 2). Relative to the control group, the low-concentration group (C vs. L) contained 507 upregulated and 452 downregulated genes. The medium-concentration group (C vs. M) contained 24 upregulated and 35 downregulated genes. In the high-dose survivor phenotype (C vs. HL), 69 genes were upregulated and 37 downregulated, whereas the high-dose dead phenotype (C vs. HD) contained 131 upregulated and 35 downregulated genes. Thus, the largest transcriptional shift was observed in the lethal phenotype, whereas the medium-dose group showed the fewest DEGs among the four contrasts.

Because DEG numbers did not increase monotonically with nominal concentration, we did not interpret DEG counts alone as a linear proxy for exposure severity. Instead, we focused on comparison-specific functional enrichment patterns and cross-omics concordance. In addition, sequencing depth, sample-to-sample distance, and replicate-bucket distribution were checked to reduce the likelihood that the observed DEG pattern arose from obvious technical bias or bucket-driven clustering.

#### 3.1.3. Gene Ontology (GO) Functional Enrichment

**Figure 3:**
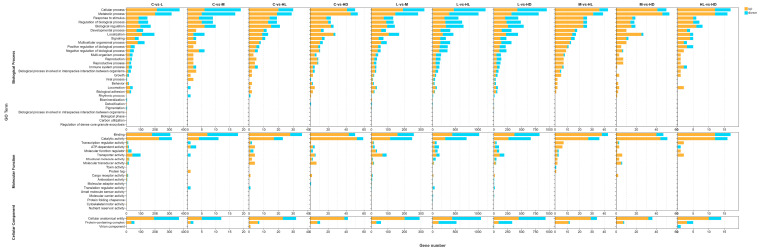
GO enrichment analysis of differentially expressed genes in control-based comparisons. GO enrichment results are shown for the four main comparisons: Enriched terms are grouped into the three Gene Ontology domains: biological process, molecular function, and cellular component. The terms shown represent the major functional categories identified among differentially expressed genes in each comparison.

GO enrichment analysis indicated that acute glyphosate exposure affected multiple biological process, molecular function, and cellular component categories, although the dominant functional signals differed among comparisons (Figure 3). In the C vs. L comparison, enriched terms were mainly associated with metabolic processes, lipid-related functions, and membrane-associated activities, suggesting early metabolic adjustment in the intestine under low-dose exposure. In the C vs. M comparison, the enriched categories shifted toward immune-related and stress-responsive functions, indicating the recruitment of host defense and stress signaling pathways at the medium dose. In the C vs. HL comparison, the enriched GO profile was narrower and more consistent with regulatory and protein-handling processes. The broadest enrichment pattern was observed in the C vs. HD comparison, where proteostasis-related functions, immune-associated categories, and apoptosis-related processes were simultaneously represented, consistent with extensive intestinal functional disruption in the lethal phenotype.

#### 3.1.4. KEGG Pathway Enrichment Highlights Comparison-Specific Functional Shifts

**Figure 4:**
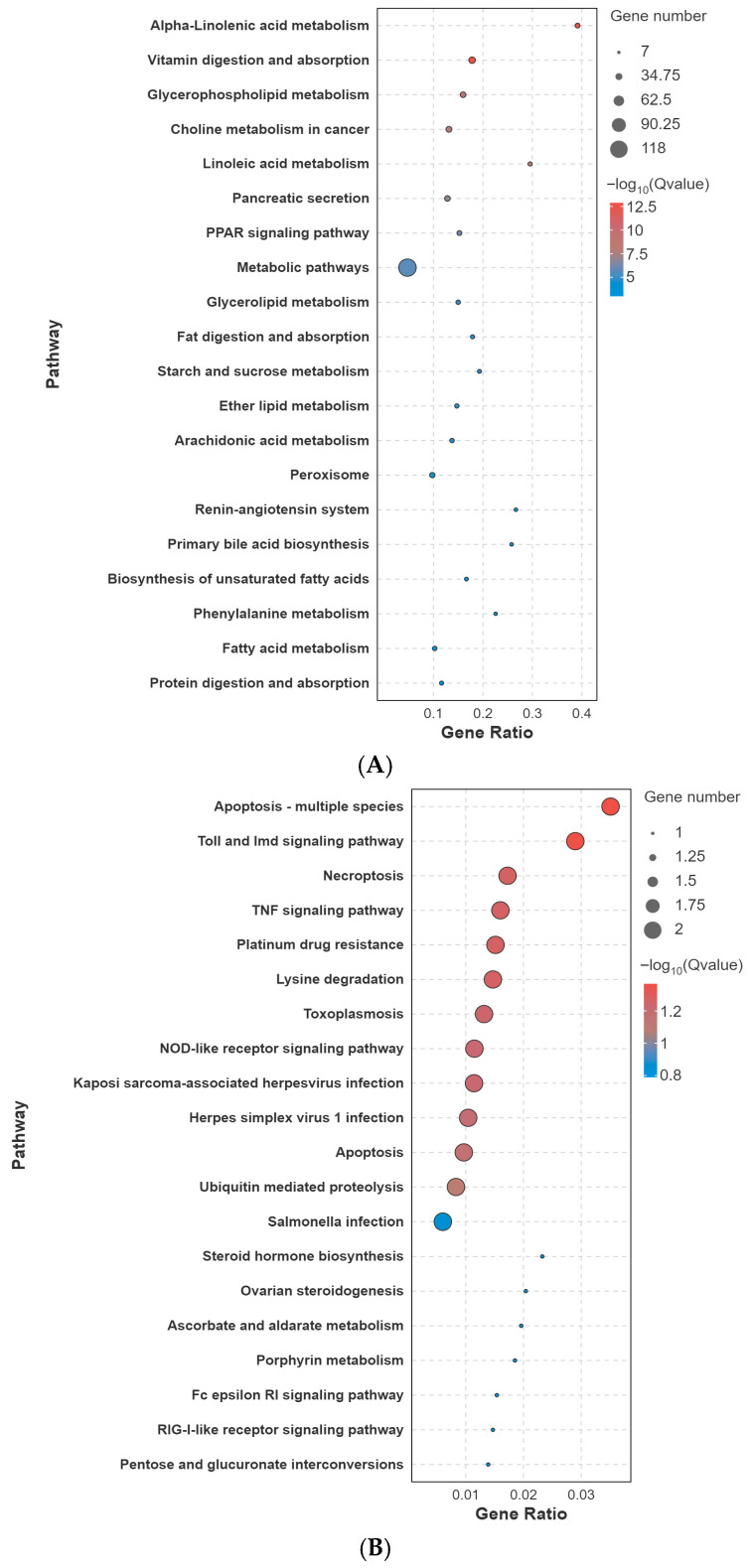

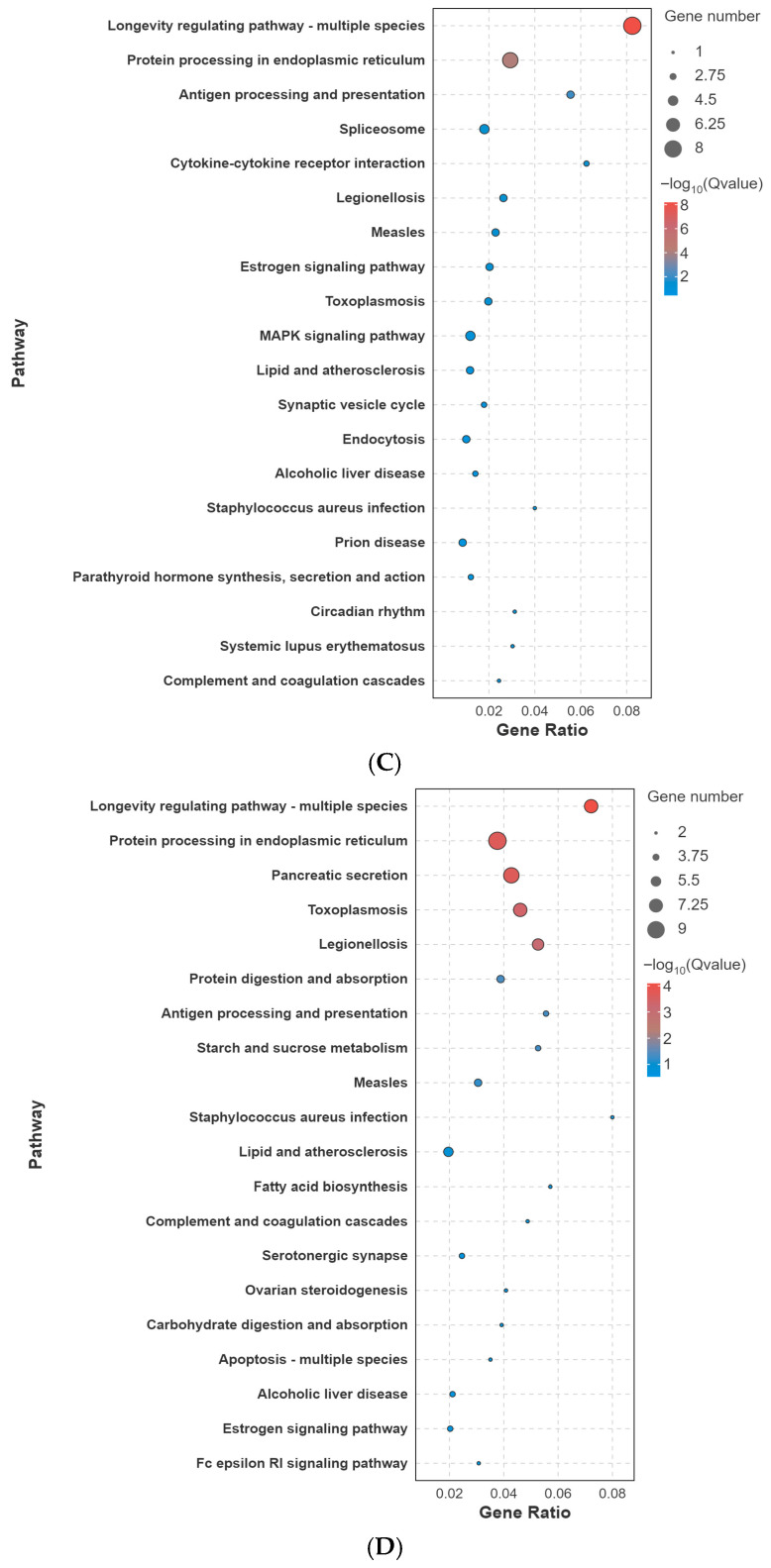
KEGG pathway enrichment of differentially expressed genes in control-based comparisons. Bubble plots show the major enriched KEGG pathways for (**A**) C vs. L, (**B**) C vs. M, (**C**) C vs. HL, and (**D**) C vs. HD. Bubble size indicates the number of differentially expressed genes mapped to each pathway, the x-axis indicates the gene ratio, and bubble color represents enrichment significance as –log10(q-value). Here, q-value refers to the FDR-adjusted *p*-value.

KEGG pathway enrichment further supported comparison-specific transcriptomic remodeling under glyphosate exposure (Figure 4). In the C vs. L comparison, enriched pathways were mainly related to lipid metabolism and digestion/absorption, including alpha-linolenic acid metabolism, linoleic acid metabolism, arachidonic acid metabolism, glycerophospholipid and glycerolipid metabolism, vitamin digestion and absorption, fat digestion and absorption, and starch and sucrose metabolism. Additional enrichment in PPAR signaling and peroxisome pathways suggested early metabolic reorganization linked to lipid handling and redox-related metabolism.

In the C vs. M comparison, the enriched pathways shifted toward immune and cell fate regulation, including apoptosis, necroptosis, TNF signaling, Toll and Imd signaling, NOD-like receptor signaling, cytokine–cytokine receptor interaction, and ubiquitin-mediated proteolysis. This pattern suggests that, although the number of DEGs was relatively small, the affected genes were concentrated in stress- and immunity-related pathways.

In the C vs. HL comparison, enriched pathways were more strongly associated with proteostasis and regulatory functions, including the longevity regulating pathway, protein processing in the endoplasmic reticulum, spliceosome, antigen processing and presentation, circadian rhythm, and endocytosis. In the C vs. HD comparison, protein processing in the endoplasmic reticulum and the longevity regulating pathway remained prominent, but the enrichment spectrum broadened to include digestive/secretory and immune–death-related modules, such as pancreatic secretion, protein digestion and absorption, carbohydrate digestion and absorption, complement and coagulation cascades, antigen processing and presentation, and apoptosis. Overall, the KEGG results indicate that glyphosate exposure induced comparison-specific transcriptomic reprogramming, with the most extensive intestinal disruption observed in the lethal phenotype.

### 3.2. Metabolomic Response to Acute Glyphosate Exposure

#### 3.2.1. Metabolomic Profiling and Multivariate Discrimination

**Figure 5:**
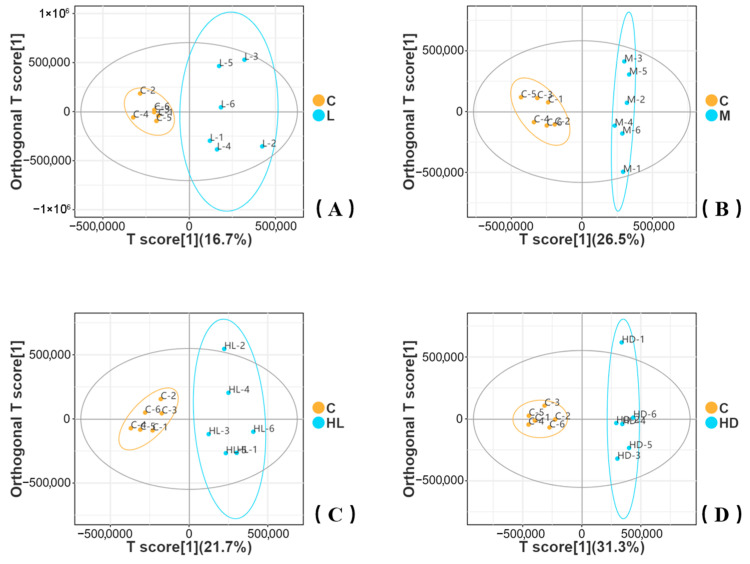

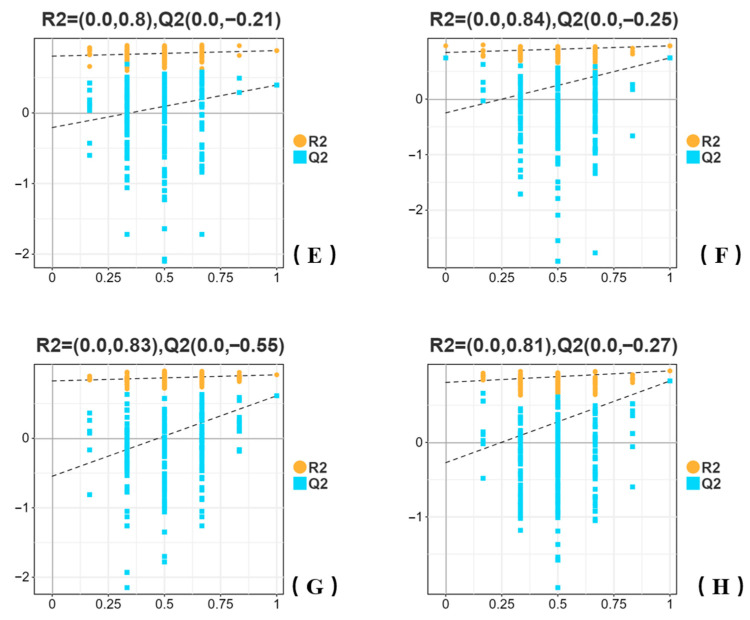
OPLS-DA discrimination of intestinal metabolomes and permutation validation across glyphosate exposure groups. (**A**–**D**) OPLS-DA score plots for pairwise comparisons between the control group (**C**) and exposed groups: (**A**) C vs. L, (**B**) C vs. M, (**C**) C vs. HL, and (**D**) C vs. HD. The x-axis (T score) represents the sample score on the predictive latent component of the OPLS-DA model. Ellipses indicate the 95% confidence region for each group. (**E**–**H**) Permutation tests for the corresponding OPLS-DA models in (**A**–**D**). In these plots, R2 represents the goodness of fit of the model, and Q2 represents its predictive ability. Differential metabolites were screened using VIP > 1 and *p* < 0.05. A relatively low or negative Q^2^ in permutation testing indicates limited predictive ability of the corresponding model and warrants cautious interpretation.

Untargeted LC–MS/MS analysis revealed clear metabolomic responses to acute glyphosate exposure. Differential metabolites were screened using VIP > 1 and *p* < 0.05. OPLS-DA score plots showed separation between the control group and each exposed group across the four main comparisons (C vs. L, C vs. M, C vs. HL, and C vs. HD) (Figure 5A–D). The separation along the predictive component increased from the low- to medium-dose comparison and was most pronounced in the high-dose dead phenotype (HD), whereas the high-dose survivor phenotype (HL) displayed an intermediate pattern.

Permutation validation was used to assess OPLS-DA model robustness (Figure 5E–H). In these plots, R2 represents model goodness of fit, and Q2 represents predictive ability. The score plot axis label “T score” refers to the sample score on the predictive latent component. Model performance was stronger in C vs. M, C vs. HL, and C vs. HD, whereas the C vs. L comparison showed weaker predictability and was therefore interpreted more cautiously. A relatively large discrepancy between R^2^ and Q^2^ in the permutation results indicates that, although some models captured group structure in the observed data, their predictive performance under cross-validation was limited. In addition, the mostly negative Q^2^ intercepts in the permutation plots are consistent with weak predictive ability in the permuted models and therefore do not support overinterpretation of these supervised models as robust predictors, particularly for the C vs. L comparison. Accordingly, the OPLS-DA results were interpreted as supportive multivariate evidence and were considered together with pathway enrichment and other downstream analyses.

#### 3.2.2. Distribution of Differential Metabolites Across KEGG Pathway Classes

**Figure 6:**
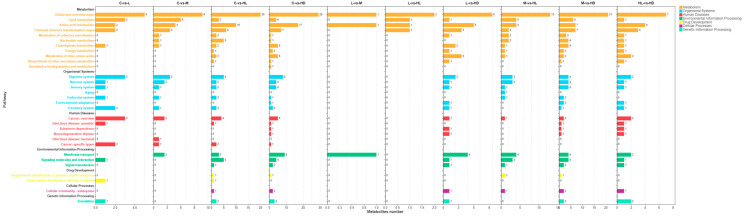
Distribution of differential metabolites across KEGG pathway classes in pairwise comparisons. Bars indicate the numbers of differential metabolites assigned to different KEGG pathway classes across comparisons among C, L, M, HL, and HD. Differential metabolites were defined using VIP > 1 and *p* < 0.05.3.2.3. KEGG pathway enrichment of differential metabolites.

The numbers and functional classes of differential metabolites varied markedly among comparisons (Figure 6), indicating both dose- and phenotype-dependent metabolic remodeling. Comparisons involving the lethal phenotype (especially C vs. HD, M vs. HD, and L vs. HD) contained the largest numbers of pathway-assigned differential metabolites, whereas comparisons among non-lethal exposure states showed fewer pathway-mapped changes. Across comparisons, the most strongly represented KEGG classes belonged to metabolism, especially global and overview maps, lipid metabolism, and amino acid metabolism, suggesting broad shifts in energy supply, membrane turnover, and nitrogen metabolism. In addition, categories related to organismal systems and environmental information processing, including the digestive system, nervous system, membrane transport, signaling molecules and interaction, and signal transduction, were more frequently represented in comparisons involving HD. Overall, the pathway-class distribution supports a progressive broadening of intestinal metabolic disturbance with increasing exposure severity, with the most pronounced redistribution occurring in the lethal phenotype.

**Figure 7:**
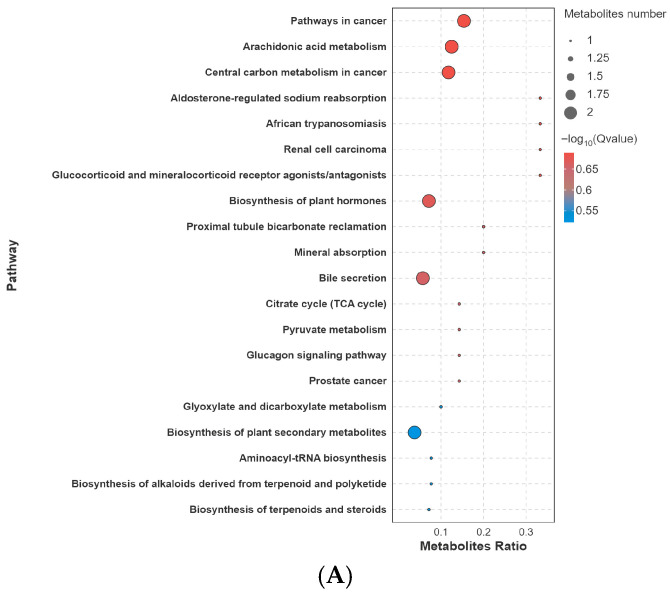

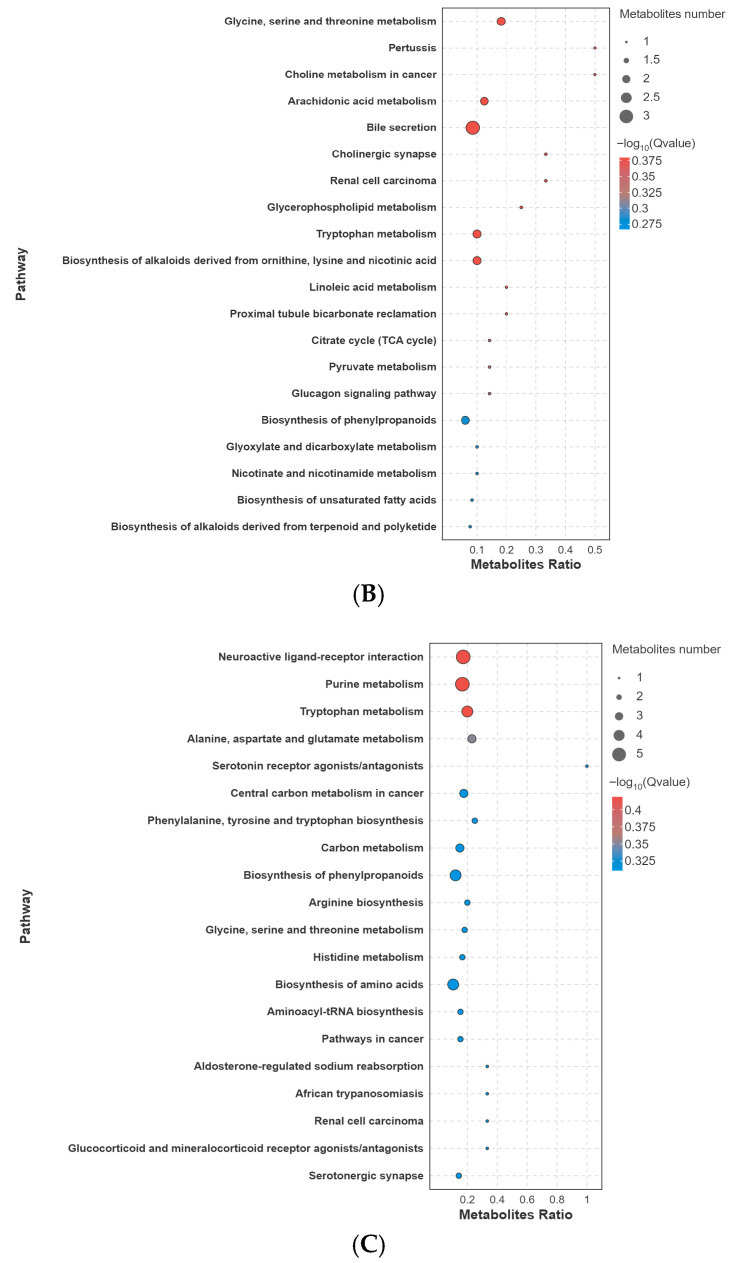

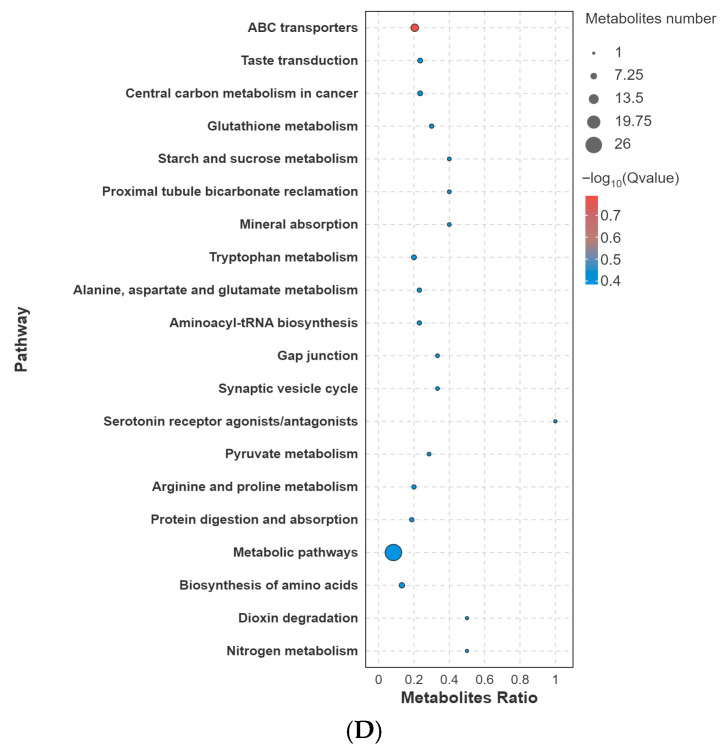
KEGG pathway enrichment of differential metabolites in control-based comparisons. Enriched metabolic pathways are shown for (**A**) C vs. L, (**B**) C vs. M, (**C**) C vs. HL, and (**D**) C vs. HD. The results illustrate dose- and phenotype-dependent metabolic remodeling in the intestine following acute glyphosate exposure.

KEGG pathway enrichment analysis showed that acute glyphosate exposure induced dose- and phenotype-dependent remodeling of intestinal metabolic pathways (Figure 7). In the C vs. L comparison, enriched pathways were mainly associated with lipid mediator metabolism and basal energy metabolism, including arachidonic acid metabolism, bile secretion, pyruvate metabolism, and the TCA cycle. In the C vs. M comparison, enrichment broadened to include amino acid and membrane lipid pathways, such as glycine, serine and threonine metabolism, tryptophan metabolism, and glycerophospholipid metabolism, while core carbon metabolism remained represented.

In the high-dose survivor phenotype (C vs. HL), enriched pathways included neuroactive ligand–receptor interaction, serotonergic synapse, tryptophan metabolism, alanine/aspartate/glutamate metabolism, and purine metabolism, suggesting phenotype-associated metabolic adjustment involving neuroactive and nitrogen-related processes. The lethal phenotype (C vs. HD) showed the broadest pathway enrichment, including glutathione metabolism, ABC transporters, and starch and sucrose metabolism, consistent with marked disruption of redox homeostasis, transport regulation, and nutrient-associated metabolism under severe exposure.

### 3.3. Integrated Transcriptomic and Metabolomic Analysis

#### 3.3.1. Joint KEGG Pathway Co-Annotation of DEGs and Differential Metabolites

Joint KEGG pathway co-annotation was used to identify pathways supported by both DEGs and differential metabolites (Table 1). At the low dose (C vs. L), only one broad pathway class, metabolic pathways (ko01100), was jointly mapped, and enrichment was not supported after correction (gene q = 0.735; metabolite q = 0.333). At the medium dose (C vs. M), three pathways were co-annotated, but none remained significant after multiple-testing correction at either the gene or metabolite level, indicating a relatively weak and dispersed cross-omics signal.

In contrast, the high-dose survivor phenotype (C vs. HL) showed the clearest cross-omics convergence, with starch and sucrose metabolism (ko00500) displaying significant gene-level enrichment (gene q = 9.49 × 10^−4^), although metabolite-level significance was not retained after correction. In the lethal phenotype (C vs. HD), several pathways were jointly mapped, especially lipid-related pathways such as alpha-linolenic acid metabolism and glycerophospholipid metabolism, but none showed strong joint enrichment after correction. These results suggest that cross-omics coordination became more structured at high exposure, especially in HL, but remained distributed across multiple pathways in HD rather than concentrated in a single strongly enriched joint pathway.

#### 3.3.2. O2PLS Analysis of Integrated Data

**Figure 8:**
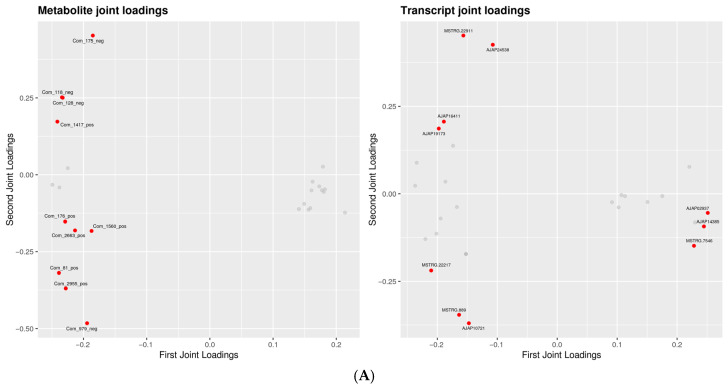

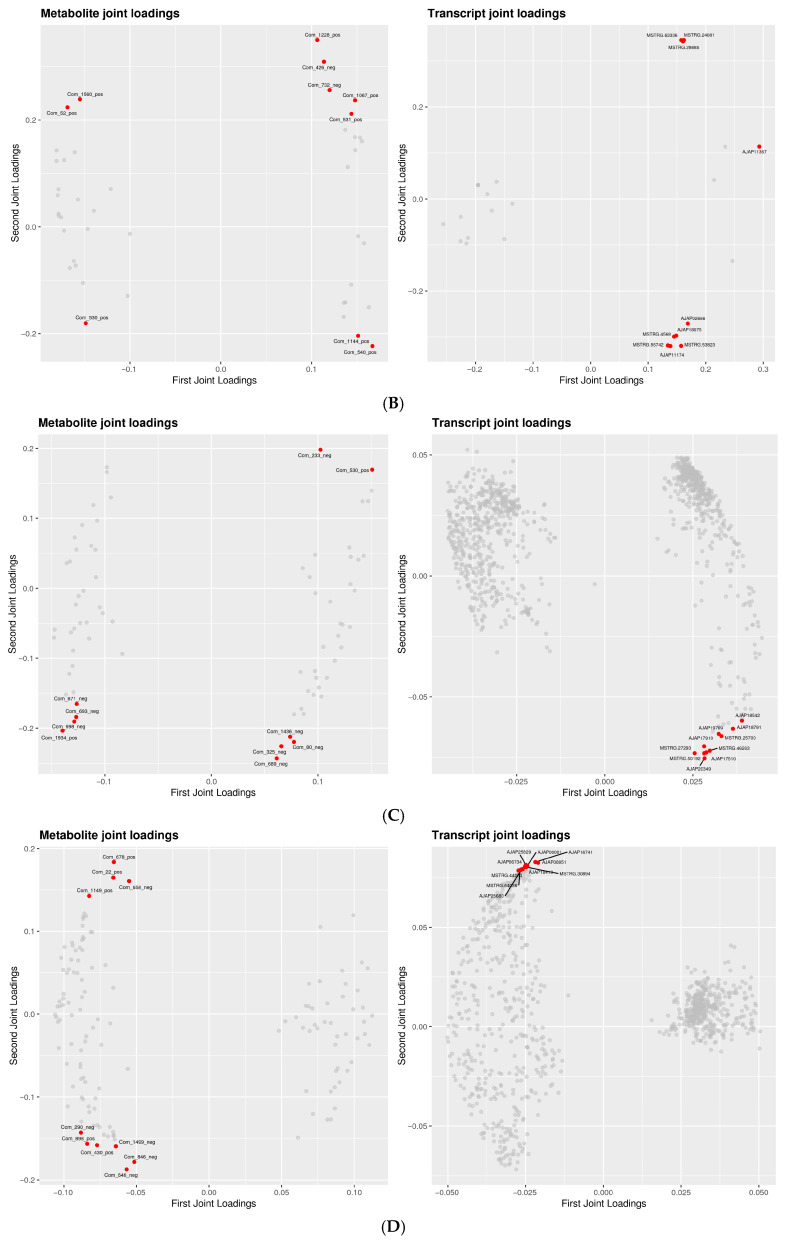
O2PLS joint loading plots of integrated transcriptomic and metabolomic data. O2PLS joint loading plots are shown for (**A**) C vs. L, (**B**) C vs. M, (**C**) C vs. HL, and (**D**) C vs. HD. These plots visualize the shared variation structure between transcriptomic and metabolomic datasets and highlight the degree of cross-omics coordination under different exposure conditions. Gray circles indicate individual genes (**right**) or metabolites (**left**).

O2PLS analysis was used to evaluate shared variation between transcriptomic and metabolomic datasets (Figure 8). At the low dose (C vs. L), discriminant metabolites were shifted mainly along the first joint component, whereas transcripts showed a more heterogeneous distribution, indicating relatively weak and dispersed cross-omics coupling. In the C vs. M and C vs. HL comparisons, the joint structure became more organized, with metabolites and transcripts showing clearer module-like separation. The C vs. HD comparison displayed the most compact and coherent joint loading pattern, suggesting the strongest transcript–metabolite covariation under the lethal phenotype. Overall, the O2PLS results support a dose- and phenotype-dependent strengthening of cross-omics coordination.

#### 3.3.3. Correlation Heatmap and Network Analysis of Transcript–Metabolite Coupling

**Figure 9:**
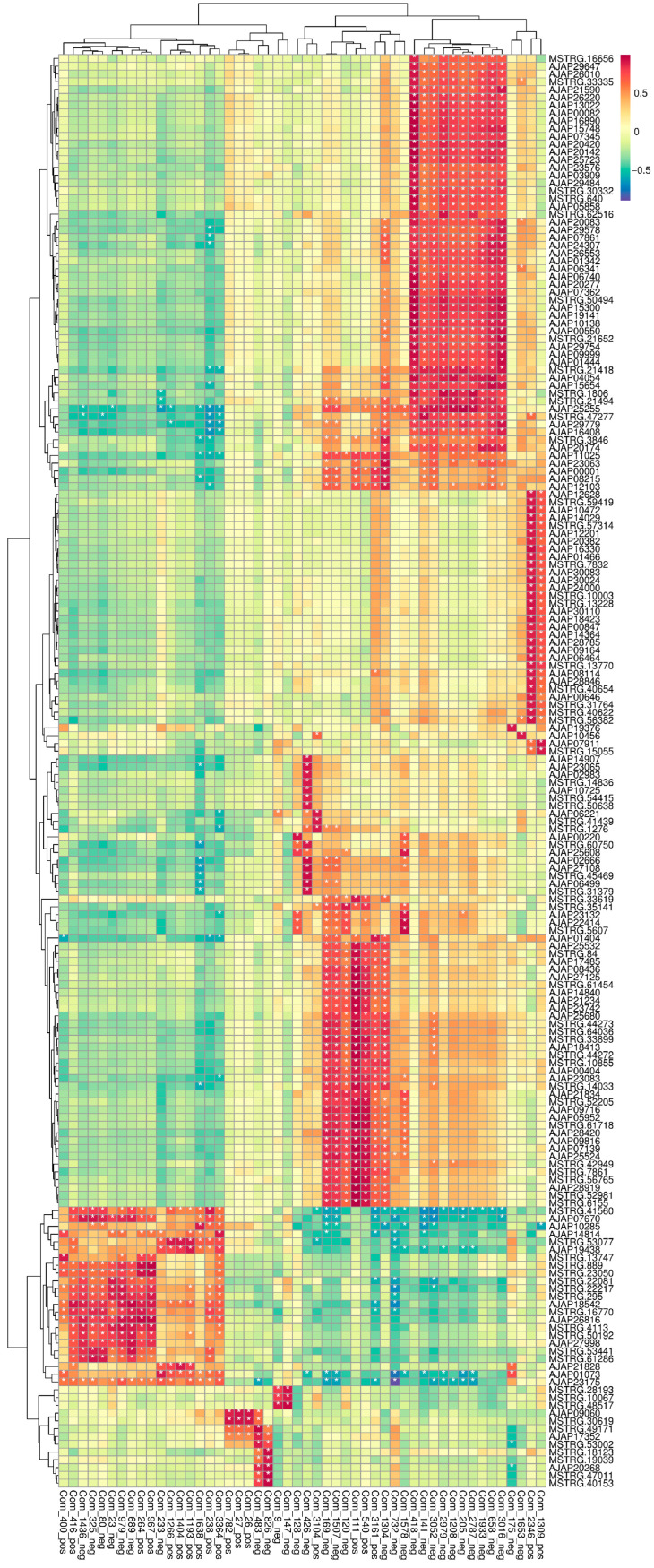
Pearson correlation heatmap of differentially expressed genes and differential metabolites. The heatmap shows Pearson correlation coefficients between all DEGs (union across comparisons) and all differential metabolites (union across comparisons). Warmer and cooler colors indicate positive and negative correlations, respectively. * indicates significant differences.

**Figure 10:**
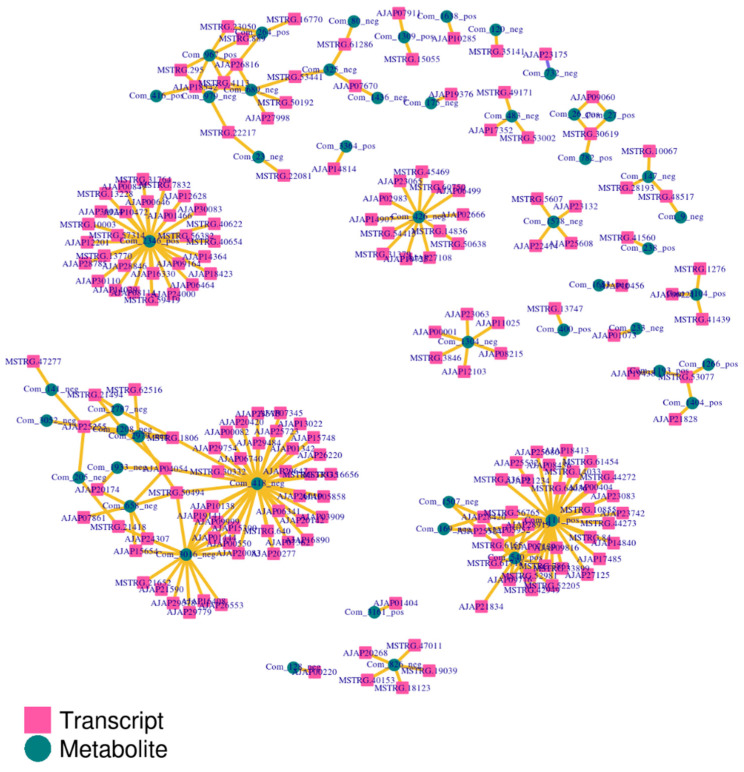
DEG–metabolite association network based on Pearson correlation analysis. The network was constructed using the top gene–metabolite pairs that met the predefined filtering criteria described in Section 2. Transcript nodes are shown as squares and metabolite nodes as circles. Edges represent significant correlations, and the modular structure reflects coordinated transcript–metabolite associations under glyphosate exposure.

To further characterize cross-omics relationships, Pearson correlations were calculated between all DEGs and all differential metabolites identified across comparisons. The resulting correlation heatmap (Figure 9) showed broad transcript–metabolite co-variation, indicating coordinated molecular responses under glyphosate exposure. Based on the predefined filtering criteria described in the Methods, a DEG–metabolite association network was then constructed (Figure 10). In this network, transcripts are shown as squares and metabolites as circles. The network exhibited a modular structure with several densely connected clusters, and many transcript nodes converged on a smaller number of metabolite nodes. Metabolites generally showed higher connectivity than transcripts, suggesting that a subset of metabolites acted as network hubs associated with multiple transcriptional changes. Together, these results support the interpretation that glyphosate exposure induced a structured multi-omics response in which transcriptomic shifts were coupled to metabolite-centered association modules.

## 4. Discussion

### 4.1. Dose- and Phenotype-Associated Intestinal Remodeling at the Transcriptomic Level

Our transcriptomic results indicate that acute glyphosate exposure induced comparison-specific intestinal remodeling in *A. japonicus*, rather than a simple monotonic increase in the number of differentially expressed genes with nominal concentration. In particular, the low-dose group showed extensive transcriptional changes enriched mainly in metabolic and digestion-related processes, whereas the medium-dose group, despite containing fewer DEGs, displayed clearer enrichment of immune-, stress-, and cell fate-related pathways. At the highest exposure level, the survivor (HL) and dead (HD) phenotypes diverged further, with HL showing stronger signatures of proteostasis and regulatory adjustment, while HD exhibited the broadest functional disruption. These patterns suggest that transcriptomic response structure, rather than DEG number alone, provides a more informative basis for interpreting exposure severity in this acute model.

This interpretation is broadly consistent with previous studies showing that glyphosate and glyphosate-based herbicides can induce transcriptomic and physiological disturbances in aquatic organisms, including oxidative stress, metabolic dysregulation, and stress-response signaling [36,37,38,39]. In this context, our results extend these observations to a marine deposit-feeding invertebrate and indicate that intestinal transcriptomic responses to acute waterborne glyphosate exposure are both dose-associated and phenotype-structured.

### 4.2. Dose- and Phenotype-Associated Intestinal Metabolic Remodeling

The metabolomic results indicate that acute glyphosate exposure induced broad but comparison-specific remodeling of intestinal metabolism in *A. japonicus*. At the low dose, enriched pathways were mainly associated with lipid mediator metabolism and basal energy metabolism, including arachidonic acid metabolism, bile secretion, pyruvate metabolism, and the TCA cycle, suggesting an early shift in membrane-related and energy-producing processes. At the medium dose, the enrichment pattern expanded toward amino acid and phospholipid metabolism, including tryptophan metabolism, glycine/serine/threonine metabolism, and glycerophospholipid metabolism, indicating a broader reorganization of nitrogen metabolism and membrane turnover. At the highest exposure level, the survivor (HL) and dead (HD) phenotypes diverged further, with HL showing stronger enrichment of neuroactive and amino acid-related pathways, whereas HD exhibited the broadest pathway spectrum, including glutathione metabolism, ABC transporters, and starch and sucrose metabolism. These findings suggest that intestinal metabolic remodeling became increasingly structured with exposure severity and was most extensive in the lethal phenotype.

Taken together, the metabolomic data support the view that glyphosate exposure disturbs not only general energy metabolism but also redox regulation, membrane turnover, and nutrient-associated intestinal functions. This broad metabolic redistribution is consistent with the transcriptomic evidence that severe exposure does not simply amplify a single response axis but instead reorganizes multiple functional modules simultaneously [36,37].

### 4.3. Cross-Omics Integration Reveals Structured Coordination of Intestinal Responses

The integrated analyses indicate that acute glyphosate exposure induced coordinated, but comparison-specific, cross-omics responses in the intestine of *A. japonicus*. Joint KEGG pathway mapping showed relatively weak and dispersed transcript–metabolite convergence at low and medium exposure levels, whereas stronger cross-omics coordination emerged at the highest exposure level. Notably, the high-dose survivor phenotype (HL) showed the clearest pathway-level convergence, particularly in starch and sucrose metabolism, while the lethal phenotype (HD) exhibited a broader but more distributed pattern of joint pathway mapping. This suggests that severe exposure did not necessarily concentrate the response into a single dominant pathway but instead generated a wider reorganization across multiple interacting functional modules.

The O2PLS results further supported this interpretation. Compared with the low-dose condition, the medium-dose and high-dose comparisons showed increasingly structured shared variation between transcriptomic and metabolomic datasets, with the most coherent joint loading pattern observed in HD. Likewise, the Pearson-based heatmap and network analysis showed that many transcriptomic changes converged onto a smaller set of highly connected metabolites, indicating that metabolite-centered association modules may represent an important organizational feature of the acute intestinal response. However, these integrative models should be interpreted as frameworks for identifying coordinated molecular patterns rather than as direct evidence of causal regulation [37].

This interpretation is broadly consistent with previous work showing that glyphosate exposure can induce coordinated multi-layer molecular responses in aquatic organisms. In red swamp crayfish, integrative transcriptomic, metabolomic, and proteomic analyses demonstrated that glyphosate tolerance is associated with broad molecular reorganization across functional systems [37]. More generally, aquatic glyphosate studies have repeatedly identified oxidative stress, metabolic disturbance, and regulatory disruption as recurring response themes [36,39]. In this context, our cross-omics results extend these observations to a marine deposit-feeding invertebrate and support the view that acute waterborne glyphosate exposure triggers a structured intestinal response in which transcriptional and metabolic changes become increasingly coordinated with exposure severity and phenotype divergence.

### 4.4. Environmental Relevance, Interpretive Scope, and Limitations of the Present Acute Multi-Omics Framework

Although glyphosate and AMPA have been increasingly reported in aquatic systems, including riverine, transitional, and coastal environments, measured concentrations from fully marine settings remain comparatively limited [10,11,12]. Therefore, the exposure concentrations used in the present study should not be interpreted as direct proxies for typical marine background levels. Rather, our design was intended to establish an acute waterborne exposure gradient that could resolve early intestinal responses and phenotype divergence under controlled laboratory conditions. In this sense, the present study is best viewed as a mechanistic acute-exposure investigation rather than a direct field-threshold or environmental risk assessment study.

This distinction is important for interpreting the strengths of the omics-based approach used here. Transcriptomic and metabolomic profiling can reveal coordinated early-response pathways that are not easily detected using mortality or single-endpoint assays alone, and they are therefore useful for identifying candidate mechanisms and potential biomarkers of sublethal stress. However, these methods are resource-intensive and depend strongly on experimental context, statistical filtering, and pathway annotation. They are not intended to replace routine large-scale ecotoxicological assessment, nor can pathway enrichment or latent variable integration by themselves establish direct causal regulation [36,37].

Several limitations should therefore be acknowledged. First, the present experiment used an acute exposure design under controlled laboratory conditions, whereas environmental exposure in coastal culture systems is more likely to involve lower, fluctuating, and potentially mixture-associated concentrations over longer periods. Second, although cross-omics coordination was observed, parts of the response remained distributed across multiple pathways, particularly in the lethal phenotype, which cautions against over-reliance on any single enriched pathway as a definitive mechanistic explanation. Third, the interpretation of pathway-level patterns would benefit from targeted validation of representative genes, metabolites, and physiological markers.

### 4.5. A Working Framework for Acute Intestinal Responses to Glyphosate in A. japonicus

Taken together, the present results support a working framework in which acute waterborne glyphosate exposure induces a structured intestinal response trajectory in *A. japonicus*. At lower exposure, the response is characterized mainly by metabolic and digestion-related adjustment; at intermediate exposure, the affected functions shift toward immune regulation, stress signaling, and cell fate-associated pathways; and at the highest exposure level, the divergence between surviving and dead individuals becomes a dominant feature, accompanied by broader disruption of proteostasis, redox balance, transport, and nutrient-associated metabolism. This pattern is consistent with the view that the intestinal system is not only an early target of acute glyphosate stress in this deposit-feeding marine invertebrate but also a functionally integrated interface through which phenotype divergence can emerge under severe exposure [36,37,39].

Importantly, the combination of transcriptomic, metabolomic, and cross-omics analyses adds resolution to this response trajectory by showing that severe exposure is associated not only with larger-scale molecular disturbance but also with stronger organization of transcript–metabolite relationships. In this sense, the present study does not merely identify isolated altered genes or metabolites but instead reveals a progressively coordinated intestinal response framework across exposure states. This interpretation should remain provisional and requires targeted validation, but it provides a useful mechanistic basis for future studies on glyphosate effects in marine non-target organisms under more environmentally relevant exposure regimes.

## 5. Conclusions

This study demonstrates that acute waterborne glyphosate exposure induces dose- and phenotype-associated intestinal molecular responses in the sea cucumber *Apostichopus japonicus*. By integrating transcriptomic and metabolomic analyses, we found that low-dose exposure was mainly associated with metabolic and digestion-related adjustment, whereas higher exposure levels were characterized by stronger involvement of immune regulation, stress signaling, proteostasis-related processes, and broader metabolic disturbance. At the highest exposure level, the divergence between surviving and dead individuals became a prominent feature, indicating that severe acute exposure was accompanied by marked phenotype-dependent intestinal remodeling.

Cross-omics analyses further showed that transcriptomic and metabolomic responses became increasingly coordinated with exposure severity, supporting a structured intestinal response framework rather than isolated molecular changes. These findings provide mechanistic insight into early intestinal responses to glyphosate in a marine deposit-feeding invertebrate under controlled acute exposure conditions.

Because measured glyphosate data from fully marine environments remain limited, the present results should be interpreted primarily as evidence from a mechanistic acute-exposure model rather than as a direct basis for environmental threshold assessment. Nevertheless, this study provides a useful foundation for future work aimed at validating candidate pathways and biomarkers under more environmentally relevant exposure scenarios.

## Figures and Tables

**Table 1:** Summary of joint KEGG pathway mapping of differentially expressed genes and differential metabolites across control-based comparisons. Shared pathways were identified by co-annotation of DEGs and differential metabolites to the KEGG database. Gene q-values and metabolite q-values represent FDR-adjusted *p*-values for pathway enrichment at the gene and metabolite levels, respectively.

Comparison	Key Co-Mapped Pathway (KEGG ID)	Co-Mapped Genes (*n*)	Co-Mapped Metabolites (*n*)	Gene q-Value	Metabolite q-Value
C vs. L	Metabolic pathways (ko01100)	2	3	0.735225	0.332971
C vs. M	Longevity regulating pathway–worm (ko04212)	1	1	0.146282	0.252753
C vs. M	Lysine degradation (ko00310)	1	1	0.146282	0.360103
C vs. M	Metabolic pathways (ko01100)	1	7	0.884627	0.614853
C vs. HL	Starch and sucrose metabolism (ko00500)	10	2	0.000949	0.192255
C vs. HL	ABC transporters (ko02010)	9	4	0.300750	0.352650
C vs. HD	α-Linolenic acid metabolism (ko00592)	5	1	0.194593	0.474384
C vs. HD	Glycerophospholipid metabolism (ko00564)	10	1	0.210840	0.474384
C vs. HD	Synaptic vesicle cycle (ko04721)	5	3	0.999997	0.342880

## Data Availability

The raw sequencing data have been deposited in the NCBI Sequence Read Archive (SRA) database under BioProject ID PRJNA1439585. The data are currently associated with temporary submission ID SUB16069952 and are scheduled for public release on 1 April 2027.
